# Characterization of excitatory synaptic transmission in the anterior cingulate cortex of adult tree shrew

**DOI:** 10.1186/s13041-017-0336-5

**Published:** 2017-12-18

**Authors:** Xu-Hui Li, Qian Song, Qi-Yu Chen, Jing-Shan Lu, Tao Chen, Min Zhuo

**Affiliations:** 1 Center for Neuron and Disease, Frontier Institutes of Science and Technology, Xi’an Jiaotong University, Xi’an, 710049 China; 2 Department of Anatomy & K.K. Leung Brain Research Center, Fourth Military Medical University, Xi’an, ShaanXi 710032 China; 3 Department of Physiology, Faculty of Medicine, University of Toronto, Medical Science Building, Room #3342, 1 King’s College Circle, Toronto, ON M5S 1A8 Canada

**Keywords:** Tree shrew, Glutamate, Calcium signals, Excitatory synaptic transmission, Intrinsic properties, Anterior cingulate cortex

## Abstract

The tree shrew, as a primate-like animal model, has been used for studying high brain functions such as social emotion and spatial learning memory. However, little is known about the excitatory synaptic transmission in cortical brain areas of the tree shrew. In the present study, we have characterized the excitatory synaptic transmission and intrinsic properties of pyramidal neurons in the anterior cingulate cortex (ACC) of the adult tree shrew, a key cortical region for pain perception and emotion. We found that glutamate is the major excitatory transmitter for fast synaptic transmission. Excitatory synaptic responses induced by local stimulation were mediated by AMPA and kainate (KA) receptors. As compared with mice, AMPA and KA receptor mediated responses were significantly greater. Interestingly, the frequency of spontaneous excitatory postsynaptic currents (sEPSCs) and miniature excitatory postsynaptic currents (mEPSCs) in tree shrews was significantly less than that of mice. Moreover, both the ratio of paired-pulse facilitation (PPF) and the time of 50% decay for fast blockade of NMDA receptor mediated EPSCs were greater in the tree shrew. Finally, tree shrew neurons showed higher initial firing frequency and neuronal excitability with a cell type-specific manner in the ACC. Our studies provide the first report of the basal synaptic transmission in the ACC of adult tree shrew.

## Introduction

The tree shrew has a well-developed brain and central nervous system. Cumulative evidence has shown that the tree shrew is an ideal animal model for brain diseases of humans. Molecular phylogeny and whole genome sequencing analysis studies suggest that tree shrew has a close affinity to primates [1–4]. Tree shrews have higher brain-to-body mass ratio and more developed pyramidal neuron compared with rodents [5]. Particularly, some information involved in cognitive impairment during aging, such as the amyloid accumulation and somatostatin degeneration, is missing in the mice and rats but can be found in monkeys and tree shrews [6, 7].

Both animal and human studies have consistently demonstrated that the anterior cingulate cortex (ACC) plays important roles in many major brain functions such as awareness, emotion, memory, and pain [8–15]. However, there is limited information of synaptic transmission and plasticity in cortical areas obtained from primate models. Therefore, the tree shrew will be a valuable primate-like animal for the mechanism of cortical synaptic transmission and plasticity, especially in the ACC.

Glutamate is the major excitatory neurotransmitter for synaptic transmission in the central nervous system. Glutamatergic synaptic transmission is mainly mediated by three kinds of gate ionotropic receptors, α-amino-3-hydroxy-5-methyl-4-isoxazole-propionic acid (AMPA), kainate (KA) and N-methyl-D-aspartate (NMDA) receptors [8, 13, 16–18]. Integrative experimental approaches including genetic, biochemical, electrophysiological and pharmacological methods demonstrate that AMPA, KA and NMDA receptors are required for distinct physiological functions and pathological conditions in the ACC, including chronic pain, fear memory, anxiety and aversion [8, 10, 15, 19–23]. However, since previous investigations are mainly focused on glutamatergic synaptic transmission in rodents, less information is known about the synaptic transmission in the ACC of primates. Using tree shrews as a primate model, our recent studies have found that both the volume of the ACC and the sizes of cell bodies in the ACC pyramidal neurons of the tree shrew are larger than those in the mouse and rat. Furthermore, there are more apical/basal dendritic branches and apical dendritic spines of the ACC pyramidal neurons in tree shrews compared with rodents [5]. However, it is not known whether the basic excitatory synaptic transmission and intrinsic properties of pyramidal neurons in the ACC of tree shrews are different from those in rodents.

In the present study, by combing whole-cell patch recording, pharmacology blocking, and two-photon calcium imaging observation, we investigated the composition characteristics of basal synaptic transmission in the ACC pyramidal neurons of tree shrew. Using mice as a control, we found the AMPA and KA receptor mediated postsynaptic responses were enlarged, but the presynaptic glutamate release probabilities were lower in tree shrews. The global calcium signals and intrinsic neuronal excitability of pyramidal neurons were also found higher in tree shrews. Our studies provide the first report for the basic electrophysiological characteristics of glutamatergic synaptic transmission and neuronal properties in the ACC pyramidal neurons of tree shrews.

## Methods

### Animals

Experiments were performed with adult male tree shrews (10–12 months old) and male C57BL/6 mice (6–8 weeks old). Tree shrews were purchased from Kunming Institute of Zoology in China. All tree shrews and mice were maintained on a 12 h light/dark cycle with food and water provided ad libitum. Animal care, as well as all experiments, was conducted in accordance with the European Community guidelines for the use of experimental animals (86/609/EEC). All performed research protocols were approved by the Ethics Committee of Xi’an Jiaotong University.

### Brain slice preparation

Coronal brain slices (300 μm) at the level of the ACC were prepared using standard methods [22, 24–26]. Adult tree shrews and mice were anesthetized with 1–2% isoflurane. The whole brain was quickly removed from the skull and submerged in the oxygenated (95% O_2_ and 5% CO_2_), ice cold cutting artificial cerebrospinal fluid (ACSF) containing the following (in mM): 252 sucrose, 2.5 KCl, 6 MgSO_4_, 0.5 CaCl_2_, 25 NaHCO_3_, 1.2 NaH_2_PO_4_ and 10 glucose, pH 7.3–7.4. After cooling in the ACSF for a short time, the whole brain was trimmed for an appropriate part to glue onto the ice-cold stage of a vibrating tissue slicer (VT1200S, Leica). Slices were transferred to a submerged recovery chamber containing oxygenated (95% O_2_ and 5% CO_2_) ACSF (in mM): 124 NaCl, 4.4 KCl, 2 CaCl_2_, 1 MgSO_4_, 25 NaHCO_3_, 1 NaH_2_PO_4_, and 10 glucose at room temperature for recording at least 1 h later.

### Whole cell patch-clamp recording

Whole cell recordings were performed in a recording chamber on the stage of a BX51W1 (Olympus) microscope equipped with infrared differential interference contrast (DIC) optics for visualization. EPSCs were recorded from layer II/III neurons with an Axon 200B amplifier (Molecular Devices), and the stimulations were delivered by a bipolar tungsten stimulating electrode placed in layer V/VI of the ACC. The recording pipettes (3–5 MΩ) were filled with a solution containing (in mM) 145 K-gluconate, 5 NaCl, 1 MgCl_2_, 0.2 EGTA, 10 HEPES, 2 Mg-ATP, 0.1 Na^3^-GTP (adjusted to pH 7.2 with KOH, 290 mOsmol). AMPA and kainate receptor mediated EPSCs were induced by repetitive stimulations at 0.02 Hz, and neurons were voltage clamped at −60 mV in the presence of AP5 (50 μM) for AMPA currents and both AP5 and GIKI 53655 (100 μM) for KA currents. NMDA receptor mediated EPSCs were pharmacologically isolated in Mg^2+^-free ACSF containing CNQX (20 μM) and glycine (1 μM), and neurons were voltage-clamped at −20 to −30 mV and induced by repetitive stimulations at 0.05 Hz. The patch electrode internal solution (in mM) 112 Cs-Gluconate, 5 TEA-Cl, 3.7 NaCl, 0.2 EGTA, 10 HEPES, 2 Mg-ATP, 0.1 Na^3^-GTP and 5 QX-314 (adjusted to PH 7.2 with CsOH, 290 mOsmol) were used for recording NMDA receptor mediated EPSCs and AMPA, KA, NMDA receptors mediated I-V curves. For miniature EPSCs (mEPSCs) recording, TTX (1 μM) was added in the perfusion solution. The current-clamp configuration was used recording action potentials (APs) for a single spike (current injection of 100 pA/5 ms) and five spikes at 5, 10, 20, and 50 Hz (current injection five times of 100 pA/5 ms at different frequencies). Picrotoxin (100 μM) was always present to block GABA_A_ receptor mediated inhibitory synaptic currents in all experiments. Access resistance was 15–30 MΩ and monitored throughout the experiment. Data were discarded if access resistance changed 15% during an experiment. Data were filtered at 1 kHz, and digitized at 10 kHz using the digidata 1440A.

To identify the morphological properties of the pyramidal cells in the tree shrew, 0.5% biocytin was added into the recording solution for the labeled patched neurons. After recording, the brain slices containing biocytin labeled cells were immediately fixed with 4% paraformaldehyde in 0.1 M PB (pH 7.4, containing saturation picric acid) for 4 h at room temperature. Then the slices were transferred to 30% sucrose overnight at 4 °C temperature. After thoroughly washing with PBS, all slices were immunostained with FITC conjugated avidin (1:200, Jackson) for 2 h at room temperature. The immunofluorescence labeled neurons were imaged with a confocal microscope (Fluoview FV1000, Olympus, Tokyo, Japan) using the appropriate filter for FITC. Each section was imaged through the depth scan and collapsed stack using z projection generated a two-dimensional reconstruction of the labeled neurons. The photomicrograph was assembled by the software of Adobe Photoshope. Only brightness and contrast were adjusted.

### Two-photon calcium imaging

In vitro calcium imaging was performed using a two-photon laser scanning microscope (Olympus FV1000-MPE system, BX61WI microscope) based on a pulsed Ti-sapphire laser (MaiTai HP DeepSee, 690–1040 nm wavelength, 2.5 W average power, 100 fs pulse width, 80 MHz repetition rate; New Port Spectra-Physics, Santa Clara, CA, USA). The laser was focused through a × 40 water-immersion objective lens (LUMPLFL/IR40XW, N.A.: 0.8, Olympus, Tokyo, Japan) and the average power was set to <15 mW (measured under the objective). Neurons were filled with indicators via the patch pipette for 20–30 min to allow diffusion of the dye into the cells. Fluorescent imaging of Cal-520 K^+^ salt (200 μM) and Alexa594 K^+^ salt (20 μM) were separated into green and red channels by a dichroic mirror and emission filters (Chroma, Bellows Falls, VT, USA), and detected by a pair of photomultiplier tubes (Hamamatsu, Shizuoka, Japan) at 800 nm. To obtain time series of fluorescent signals from global soma images, images were collected with the following parameters [26–29]: 512 × 512 pixel images, digital zoom 3× with ×40 objective (N.A. 0.8), 2-μs pixel dwell time, 50 ms/frame for frame scan model with different recording times for different recording frames. Bidirectional scanning and line-scanning models were used to increase scan speed. Each trial was repeated at least 3 times and the mean value was collected. Fluorescence changes were quantified as increases in green fluorescence from the baseline of ΔF/F = (F-F_0_)/F_0_.

### Drugs

The chemicals and drugs used in this study were as follows: all the chemicals and drugs used in this study were obtained from Sigma (St. Louis, MO, USA), except for CNQX (20 μM), which was purchased from Tocris Cookson (Bristol, UK). All experiments were conducted in the presence of picrotoxin (100 μM) to block GABA_A_ receptor mediated inhibitory synaptic currents. Drugs were prepared as stock solutions for frozen aliquots at −20 °C. All these drugs were diluted from the stock solution to the final desired concentration in the ACSF before being applied to the perfusion solution.

### Data analysis

Data were collected and analyzed with Clampex 10.3 and Clampfit 10.3 software (Molecular Devices). The data were presented as means ± SEM. Statistical analysis of differences were tested by unpaired and paired two-tailed Student’s t-test, one-way ANOVA or two-way ANOVA (Student-Newmann-Keuls or Tukey test was used for post-hoc comparisons). In all cases, * *P* < 0.05 was considered statistically significant.

## Results

### Glutamate mediated excitatory synaptic transmission in the tree shrew

**Fig. 1:**
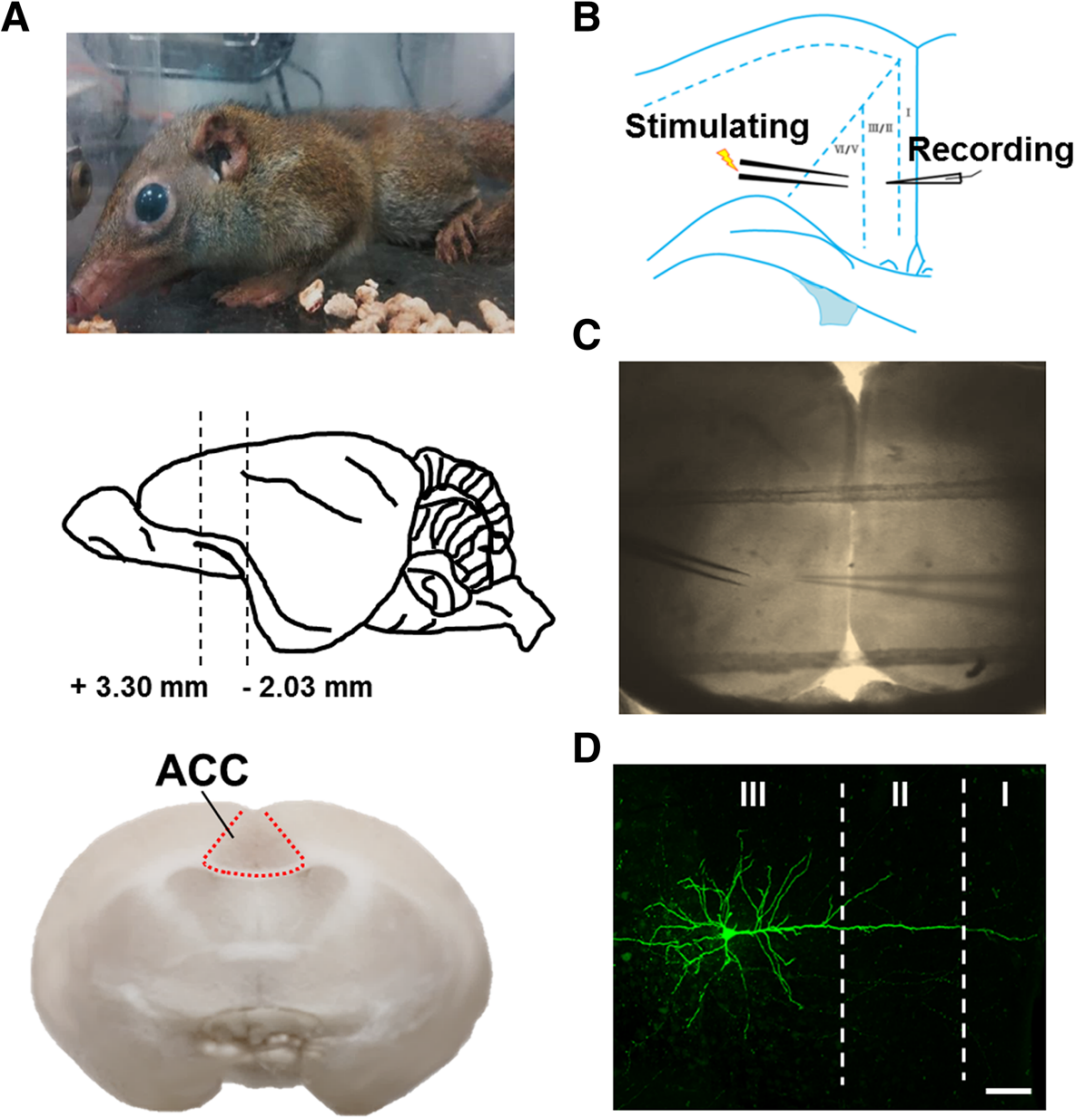
Whole cell patch-clamp recordings of layer II/III pyramidal neurons in the ACC of tree shrew. **a** Preparing process of tree shrew brain slices. Tree shrew was anesthetized with 1–2% isoflurane (upper); slices including ACC area from Bregma +3.30 to −2.03 mm (middle); representative coronal brain slice of tree shrew including ACC area (bottom). **b** and (**c**) Schematic diagram and representative recording diagram showing the placement of stimulating and recording electrodes in the ACC of tree shrew. **d** Representative photomicrograph of a biocytin-labeled pyramidal neuron in the layer II/III of ACC, scale bar: 50 μm

To explore the excitatory synaptic transmission in the ACC of the tree shrew, whole cell patch-clamp recordings were performed on pyramidal neurons in layer II/III of the ACC. In this research, there were 32 male tree shrews used in the experiments, totally. Local electrical stimulation was delivered by a bipolar stimulation electrode placed in layer V/VI of the ACC (Fig. 1b and c). Neurons in layer II/III were selected since our previous studies showed that neurons from this area receive sensory information inputs from the periphery, and play important roles in ACC related functions [8, 10, 23]. In order to characterize morphological properties of the ACC neurons, we labeled the neurons with biocytin during recording. As expected, we found that all pyramidal neurons had mass basal dendrites and a prominent apical dendrite. Basal dendrites were mainly located at same layer and surrounded the soma. Apical dendrite ascended toward the layer I with many branches (Fig. 1d).

**Fig. 2:**
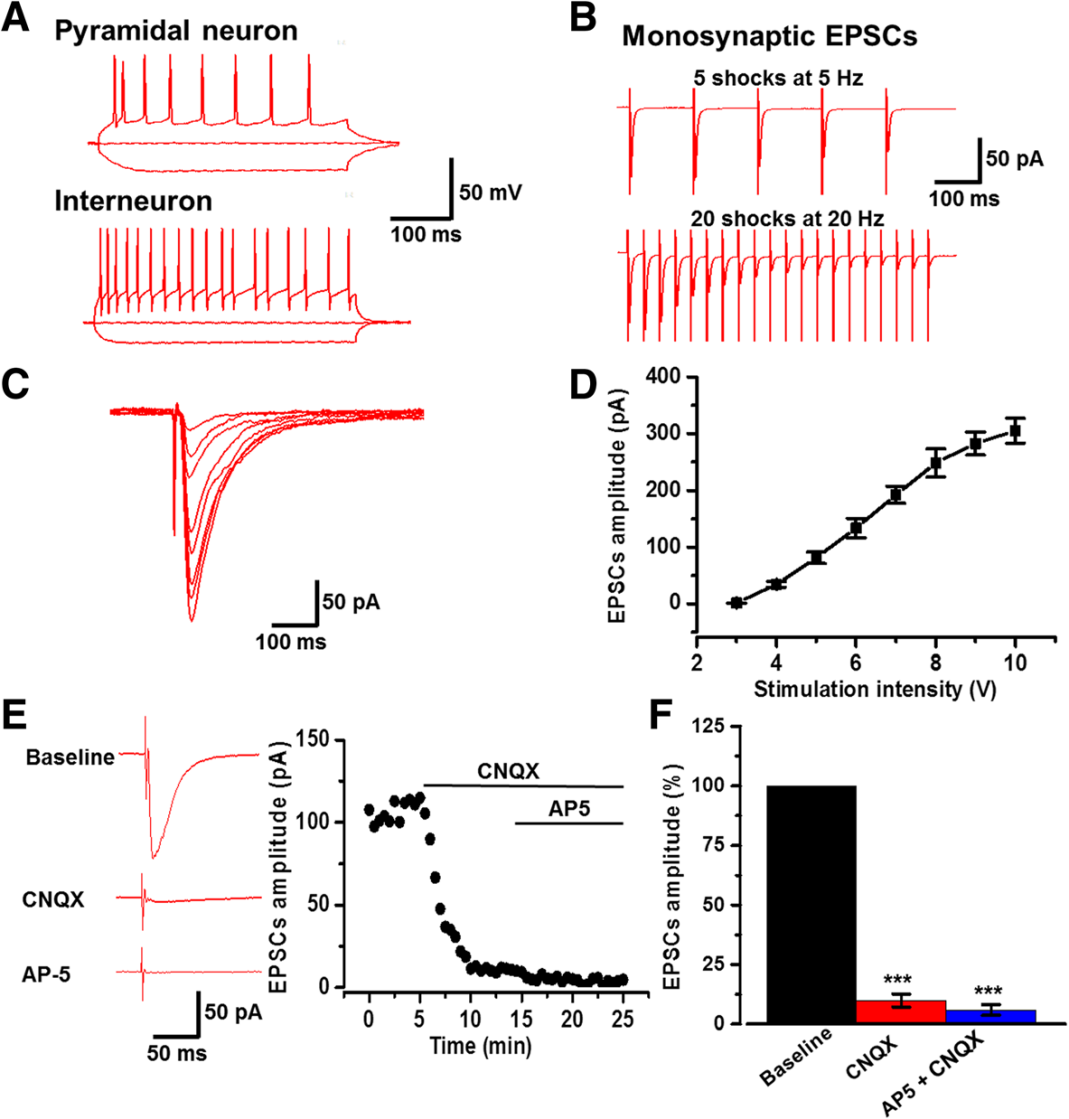
Glutamatergic neuron mediated EPSCs in the tree shrew. **a** Identification of pyramidal neuron (upper) and interneuron (bottom) by injection of step currents (−50, 0, and 50 pA). **b** Monosynaptic EPSCs induced by 5 shocks at 5 Hz (upper) and 20 shocks at 20 Hz (bottom). **c** and (**d**) Sample traces and pooled data showed the input-output relationship of basal EPSCs in the ACC of tree shrew (*n* = 7 neurons/3 tree shrews). **e** EPSCs were recorded in the presence of picrotoxin (100 μM). After the perfusion of CNQX (20 μM) 10 min, a small residual current remained that could be totally blocked by CNQX and AP5 (50 μM) together. Sample traces (left) and sample time course points (right) showed the EPSCs in the presence of CNQX and AP5. **d** Statistical results showed that the percentage of EPSCs in the presence of CNQX and AP5 (*n* = 8 neurons/4 tree shrews). ***P < 0.001, error bars indicated SEM

The pyramidal neuron of the ACC in the tree shrew was identified by injecting depolarizing currents which induced repetitive action potentials, with the firing pattern differing from interneurons (Fig. 2a) [30, 31]. Monosynaptic synaptic inputs were tested by delivering 5 shocks at 5 Hz and 20 shocks at 20 Hz (Fig. 2b). These synaptic responses followed the repetitive stimuli without failure in the presence of picrotoxin (100 μM), suggesting that they are monosynaptic in nature. To examine synaptic responses, we recorded the input (stimulation intensity)-output (EPSC amplitude) (I-O curves) relationship of excitatory postsynaptic currents (EPSCs) in the ACC neurons. We found the amplitudes of these EPSCs increased with a stimulation density dependent manner (Fig. 2c and d) (*n* = 7 neurons/3 tree shrews).

To test whether the excitatory synaptic transmission is mediated by glutamate, we bath applied an AMPA/Kainate (KA) receptor antagonist 6-cyano-7-nitroquinoxaline-2, 3-dione (CNQX, 20 μM). EPSCs were rapidly and largely reduced by CNQX. Small residual EPSCs persisted in the presence of CNQX 10 min after perfusion. These EPSCs were blocked by following application of NMDA receptor antagonist D-2-amino-5-phosphonopentanoic acid (AP5, 50 μM) (Baseline: −138.4 ± 8.5 pA; CNQX: -11.2 ± 2.0 pA, 8.1 ± 1.5% of baseline; AP5: −6.8 ± 1.4 pA, 4.9 ± 1.0% of baseline; *n* = 8 neurons/4 tree shrews; Fig. 2e and f). These results indicate that, as with the rodents, glutamate is the major excitatory synaptic transmitter in the ACC pyramidal neurons of the tree shrew and the post-synaptic responses are mainly mediated by AMPA/KA receptors, but less mediated by NMDA receptor.

### The AMPA and NMDA receptor mediated EPSCs in the tree shrew

**Fig. 3:**
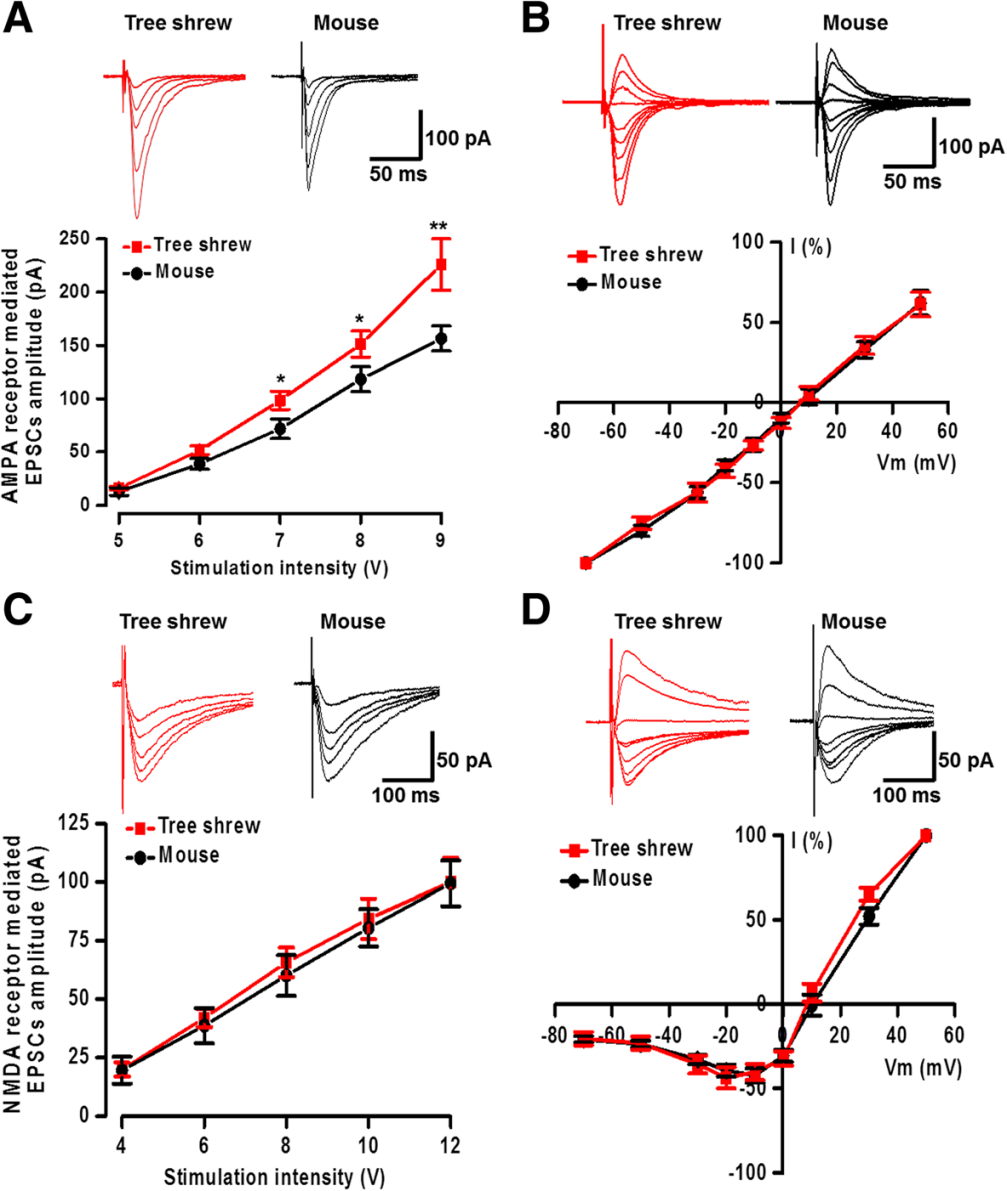
The characteristics of AMPA and NMDA receptor mediated EPSCs in the tree shrew. **a** Representative traces and pooled data showed the input-output curve of AMPA receptor mediated EPSCs were shifted to the left in tree shrew (*n* = 13 neurons/6 tree shrews) compared with in mouse (*n* = 17 neurons/6 mice). **b** AMPA receptor mediated I-V curves were not different in the ACC neurons between tree shrew and mouse (n = 8 neurons/4 tree shrews and 11 neurons/5 mice). **c** NMDA receptor mediated input-output curves in tree shrew and mouse were not different (n = 8 neurons/4 tree shrews and n = 13 neurons/4 mice). **d** NMDA receptor mediated I-V curves in tree shrew and mouse were not different between in tree shrew and mouse (n = 7 neurons/3 tree shrews and *n* = 10 neurons/4 mice). *P < 0.05, **P < 0.01, error bars indicated SEM

To investigate the properties of AMPA and NMDA receptor-mediated responses in tree shrews, the input-output responses (I-O curves) and current-voltage curves (I-V curves) were recorded in ACC neurons. Picrotoxin (100 μM) and AP5 (50 μM) were bath applied for recording AMPA receptor mediated EPSCs. As shown in Fig. 3a, we found that AMPA receptor mediated I-O curve was shifted to the left in tree shrew (*n* = 13 neurons/6 tree shrews) compared with mouse (*n* = 17 neurons/6 mice; F_(1, 151)_ = 8.24, *P* < 0.01, two-way ANOVA), indicating that the basal excitatory responses are potentiated in tree shrew. However, the I-V curves (−70 to +50 mV) were not different between tree shrew and mouse (*n* = 8 neurons/4 tree shrews and *n* = 11 neurons/5 mice; F_(1, 141)_ = 0.13, *P* = 0.72, two-way ANOVA) (Fig. 3b).

We then tested the NMDA receptor mediated responses in the tree shrew, by investigating the I-O curves and I-V curves in the presence of picrotoxin (100 μM) and CNQX (20 μM). As the results shown in Fig. 3c, NMDA receptor mediated I-O curves were not different between tree shrew and mouse (n = 8 neurons/4 tree shrews and *n* = 13 neurons/4 mice, F_(1, 95)_ = 0.26, *P* = 0.61, two-way ANOVA). Furthermore, the I-V curves were also not different between tree shrew and mouse (*n* = 7 neurons/3 tree shrews and *n* = 10 neurons/4 mice, F_(1, 127)_ = 0.49, *P* = 0.48, two-way ANOVA) (Fig. 3d). Our results suggest that the basal NMDA receptor mediated responses are not different in tree shrew and mouse.

### KA receptor mediated EPSCs in the tree shrew

**Fig. 4:**
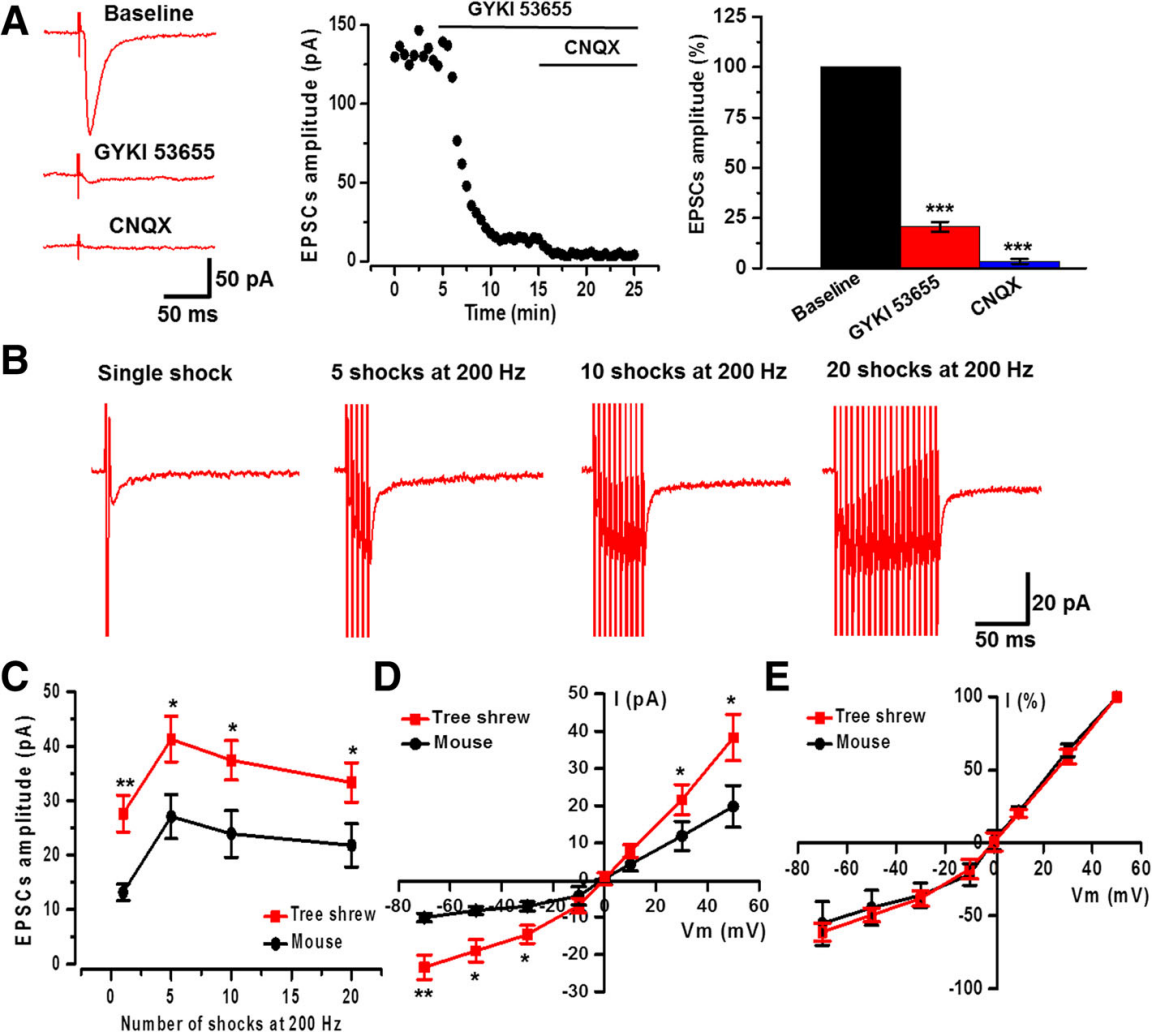
Kainate receptor mediated EPSCs in the tree shrew. **a** In the presence of picrotoxin (100 μM) and AP5 (50 μM), KA receptor mediated EPSCs could be observed after application of GYKI 53655 (100 μM) and then blocked by CNQX (20 μM). Sample traces (left), sample time course points (middle), and statistical results (right) showed that the EPSCs in the presence of GYKI 53655 and CNQX (*n* = 6 neurons/3 tree shrews). **b** Representative traces of KA receptor mediated EPSCs obtained after application of different number of stimuli (1, 5, 10 and 20 shocks) at 200 Hz. **c** Statistical results showed that the peak amplitude of the KA EPSCs in tree shrew was larger than those in mouse by repetitive stimulations (200 Hz) (*n* = 11 neurons/4 tree shrews and *n* = 9 neurons/3 mice). Note that 5 shocks induced a saturated current. The amplitude (**d**) and the percentage (**e**) of current-voltage relationship (I-V curves from −70 to +50 mV) for KA receptor mediated EPSCs in tree shrew and mouse (n = 10 neurons/4 tree shrews; n = 7 neurons/3 mice). *P < 0.05, **P < 0.01, error bars indicated SEM

In addition to AMPA receptors, KA receptors have been found to play roles in synaptic transmission in the ACC [17, 18, 23, 32]. We then examined whether KA receptors contribute to synaptic responses in the ACC neuron of tree shrew (Fig. 4). After recording a steady basal EPSCs in the presence of picrotoxin (100 μM) and AP5 (50 μM), a potent AMPA receptor antagonist GYKI 53655 (100 μM) was bath applied to isolate KA receptor mediated EPSCs. As shown in Fig. 4a, GYKI 53655 rapidly and rigorously reduced the basal EPSCs in the ACC of tree shrew. The small residual EPSCs were then blocked by following application of CNQX. As calculated, KA receptors contributed 19.4 ± 2.2% of the AMPA/KA currents (AMPA/KA EPSCs: −148.8 ± 12.7 pA; KA EPSCs: −28.8 ± 3.3 pA, *n* = 6 neurons/3 tree shrews). These results suggest that KA receptors mediate a relatively small component of the excitatory non-NMDA receptor mediated synaptic transmission in the ACC of tree shrew.

Previous studies have been shown that brief repetitive impulse trains increased KA receptor mediated EPSCs [17, 18]. To determine the summarized amplitude of KA receptor mediated EPSCs, repetitive stimuli were applied for 1, 5, 10 and 20 shocks at 200 Hz in the presence of GYKI 53655 in the ACC of tree shrew. As shown in Fig. 4b and c, the amplitudes of KA receptor mediated EPSCs were accumulated with five repetitive stimuli (−41.3 ± 4.2 pA by 5 shocks, −37.5 ± 3.6 pA by 10 shocks, and −33.4 ± 3.6 pA by 20 shocks compared with −27.6 ± 3.4 pA by single stimulation, *n* = 11 neurons/4 tree shrews, *P <* 0.05; Fig. 4c). However, the amplitudes were not further increased with more number of shocks (10–20), suggesting a saturation of the KA EPSCs. Interestingly, we found the KA receptor mediated EPSCs were significantly larger in tree shrew compared with mouse (n = 11 neurons/4 tree shrews; *n* = 9 neurons/3 mice; F_(1, 71)_ = 18.80, *P* < 0.01, two-way ANOVA) (Fig. 4c).

Next we wanted to study the further characteristics of the current-voltage (*I-V*) relationship in KA receptor mediated EPSCs. The *I-V* curve of KA receptor can reflect the calcium permeability and the subunit composition of channels [17, 18, 33]. KA EPSCs were induced by single shock in the presence of GYKI 53655. When recorded at various holding potentials ranging from −70 to 50 mV, KA EPSCs reversed at a potential of −0.12 ± 3.3 mV (*n* = 10 neurons/4 tree shrews, Fig. 4d). The mean rectification index of the KA EPSCs (ratio of estimated conductance at +40 and −60 mV) was 1.67 ± 0.17. In some cases, there were few neurons shown lower rectification index (0.93 ± 0.07, *n* = 3 neurons in total 10 neurons), indicating they have smaller outward currents of KA EPSCs. The *I-V* curves of the amplitude of KA EPSCs showed both stronger inward currents and outward currents in the ACC neuron of tree shrew than that of mouse (*n* = 10 neurons/4 tree shrews and *n* = 7 neurons/3 mice; F_(1, 112)_ = 6.07, *P* < 0.05, two-way ANOVA) (Fig. 4d). The I-V curves of the percentage of KA EPSCs were not different in the ACC neuron between tree shrew and mouse (*n* = 10 neurons/4 tree shrews and n = 7 neurons/3 mice, F_(1, 112)_ = 0.14, *P* = 0.70, two-way ANOVA) (Fig. 4e).

### Presynaptic glutamate release probability in the tree shrew

**Fig. 5:**
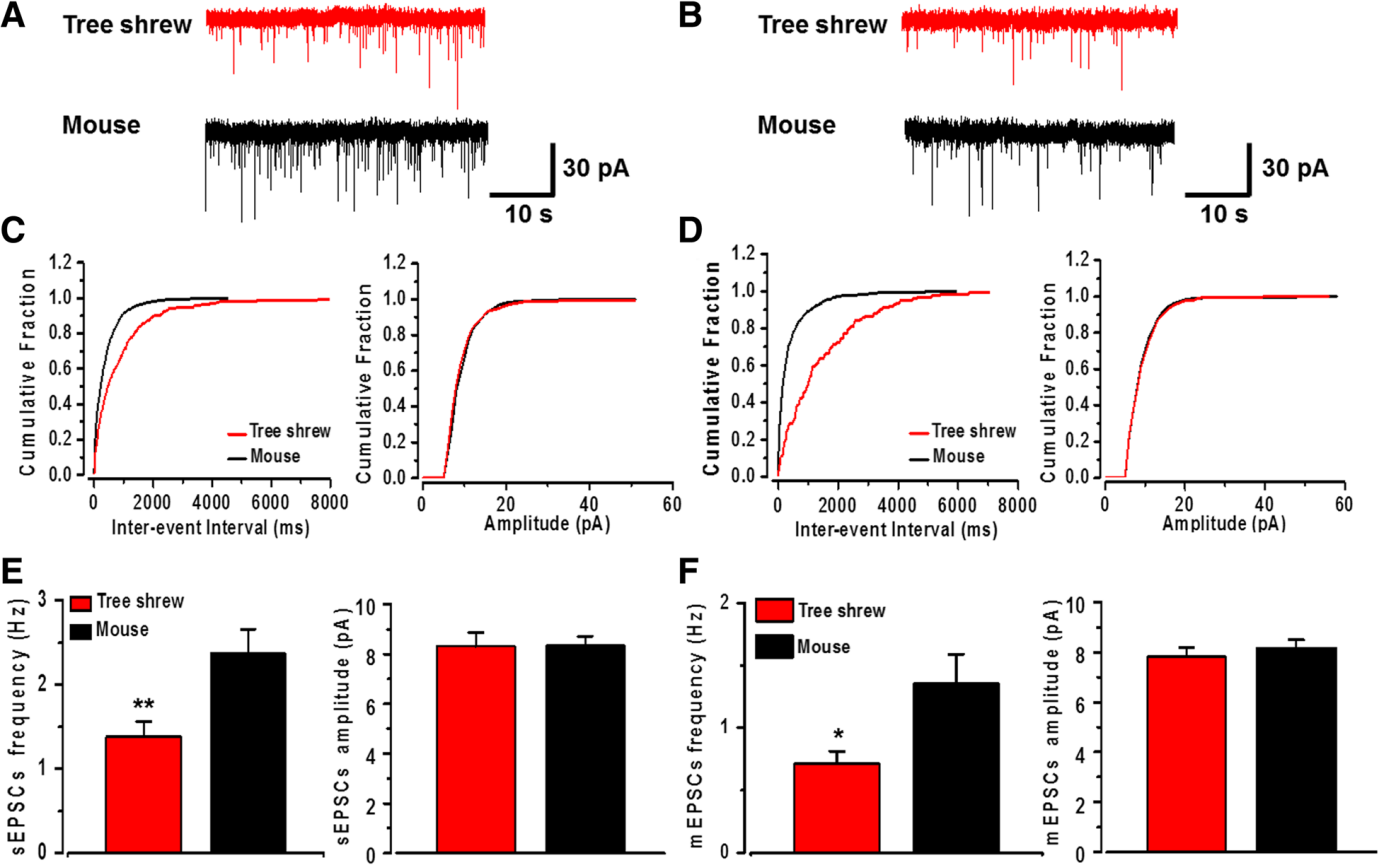
The spontaneous and miniature EPSCs in the tree shrew. **a** and (**b**) Representative traces of the sEPSCs and mEPSCs recorded in the ACC neurons of tree shrew and mouse. **c** and (**d**) Cumulative interevent interval (left) and amplitude histograms (right) of the sEPSCs and mEPSCs. **e** and (**f**) Statistical results of frequency (left) and amplitude (right) of the sEPSCs (*n* = 20 neurons/6 tree shrews and *n* = 19 neurons/5 mice) and the mEPSCs (n = 10 neurons/4 tree shrews and n = 9 neurons/3 mice). *P < 0.05, ***P < 0.001, error bars indicated SEM

To determine the presynaptic glutamate release probability in tree shrew, the spontaneous EPSCs (sEPSCs) and miniature EPSCs (mEPSCs) were recorded in the ACC neurons (Fig. 5). We found that the frequencies of sEPSCs (tree shrew: 1.38 ± 0.18 Hz, *n* = 20 neurons/6 tree shrews; mouse: 2.37 ± 0.29 Hz, *n* = 19 neurons/5 mice, *P* < 0.01) and mEPSCs (tree shrew: 0.71 ± 0.10 Hz, n = 10 neurons/4 tree shrews; mouse: 1.36 ± 0.23 Hz, *n* = 9 neurons/3 mice, *P* < 0.05) were lower in tree shrew compared with mouse. However, the amplitudes of sEPSCs (tree shrew: 8.31 ± 0.56 pA, n = 20 neurons/6 tree shrews; mouse: 8.35 ± 0.37 pA, n = 19 neurons/5 mice, *P* > 0.05) and mEPSCs (tree shrew: 7.82 ± 0.37 pA, n = 10 neurons/4 tree shrews; mouse: 8.16 ± 0.35 pA, n = 9 neurons/3 mice, *P* > 0.05) (Fig. 5) were no different. These results indicate that the presynaptic glutamate release probability is smaller in the ACC of tree shrew.

**Fig. 6:**
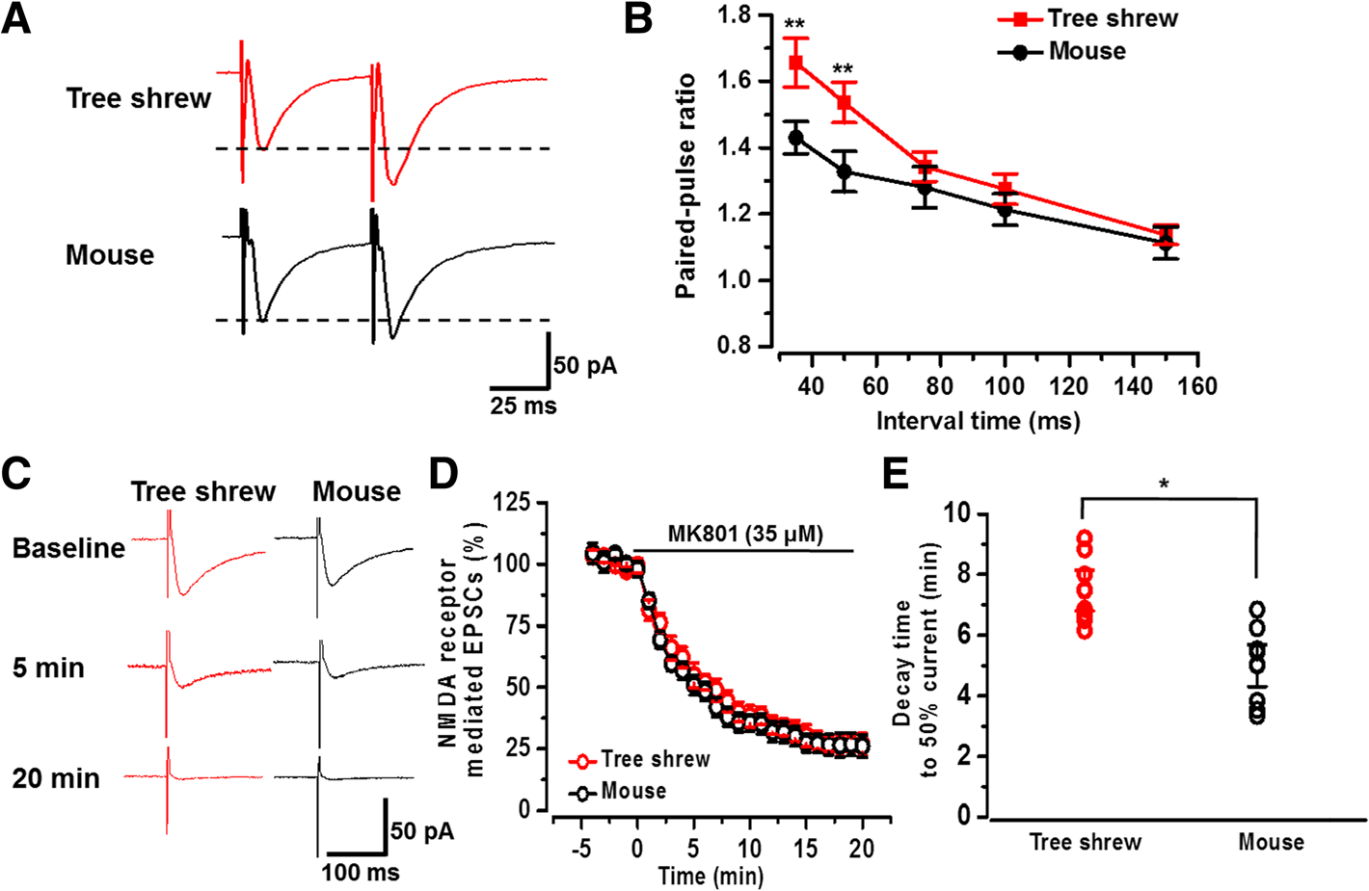
The evoked presynaptic glutamate release was decreased in the tree shrew. **a** Representative traces of paired-pulse ratio recorded at interval of 50 ms in tree shrew and mouse. **b** Statistical results showed that the paired-pulse ratio increased in tree shrew (*n* = 27 neurons/6 tree shrews) compared with mouse (*n* = 22 neurons/6 mice). **c** Representative traces of NMDA receptor mediated EPSCs at 0, 5, and 20 min in the presence of MK-801 (35 μM) in tree shrew and mouse neurons with a membrane holding at −20 mV or −30 mV. **d** Plot of time course of MK-801 blockade of NMDA receptor mediated EPSCs in tree shrew (red, n = 8 neurons/3 tree shrews) and mouse (black, n = 8 neurons/3 mice). **e** Individual and statistical data showed the decay time required for the peak amplitude of NMDA receptor mediated EPSCs to decrease to 50% of initial value in the presence of MK-801. Significantly faster time was observed in mouse than tree shrew. *P < 0.05, ***P < 0.001, error bars indicated SEM

Spontaneous and action potential related presynaptic glutamate release may come from different vesicle pools and reflect different physiological functions [34]. By testing the ratio of paired-pulse facilitation (PPF), we measured whether the electrical evoked presynaptic glutamate release is also reduced. PPF is a transient form of plasticity that is normally used to measure the presynaptic function [31]. As the results shown in Fig. 6a and b, the PPF ratios, recorded at the intervals of 35, 50, 75, 100, and 150 ms, were significantly greater in tree shrew (*n* = 27 neurons/6 tree shrews) compared with mouse (*n* = 22 neurons/6 mice) (F_(1, 227)_ = 9.78, *P* < 0.01, two-way ANOVA).

The blocking rate of NMDA receptor mediated responses by (+)-5-methyl-10,11-dihydro-5*H*-dibenzo-[*a,d*]cyclohepten-5,10-imine maleate (MK-801), a non-competitive NMDA receptor antagonist with activity-dependent manner, has been widely reported to estimate the glutamate release probability [31, 35, 36]. As shown in Fig. 6c-e, NMDA receptor mediated EPSCs were recorded in the presence of CNQX (20 μM) and picrotoxin (100 μM) at 0.1 Hz with a membrane holding at −20 or −30 mV. MK-801 (35 μM) was perfused after obtaining stable NMDA receptor mediated EPSCs. We found that MK-801 progressively blocked and completely inhibited the NMDA EPSCs in 25 min. The blocking rate of the inhibition of NMDA EPSCs by MK-801 in tree shrew was considerably slower than that of the mouse (Fig. 6d). We compared the decay time from peak to 50% value of initial amplitude of NMDA EPSCs and found the decay time in tree shrew was significantly slower than in mouse (tree shrew: 7.47 ± 0.67 min, *n* = 8 neurons/3 tree shrews; mouse: 4.97 ± 0.69 min, n = 8 neurons/3 mice, *P* < 0.05). Taken together, these results indicate that the rate of presynaptic glutamate release in the ACC of tree shrews is slower as compared with that in mice.

### Stimulation intensity and frequency dependent global calcium signals in the tree shrew

**Fig. 7:**
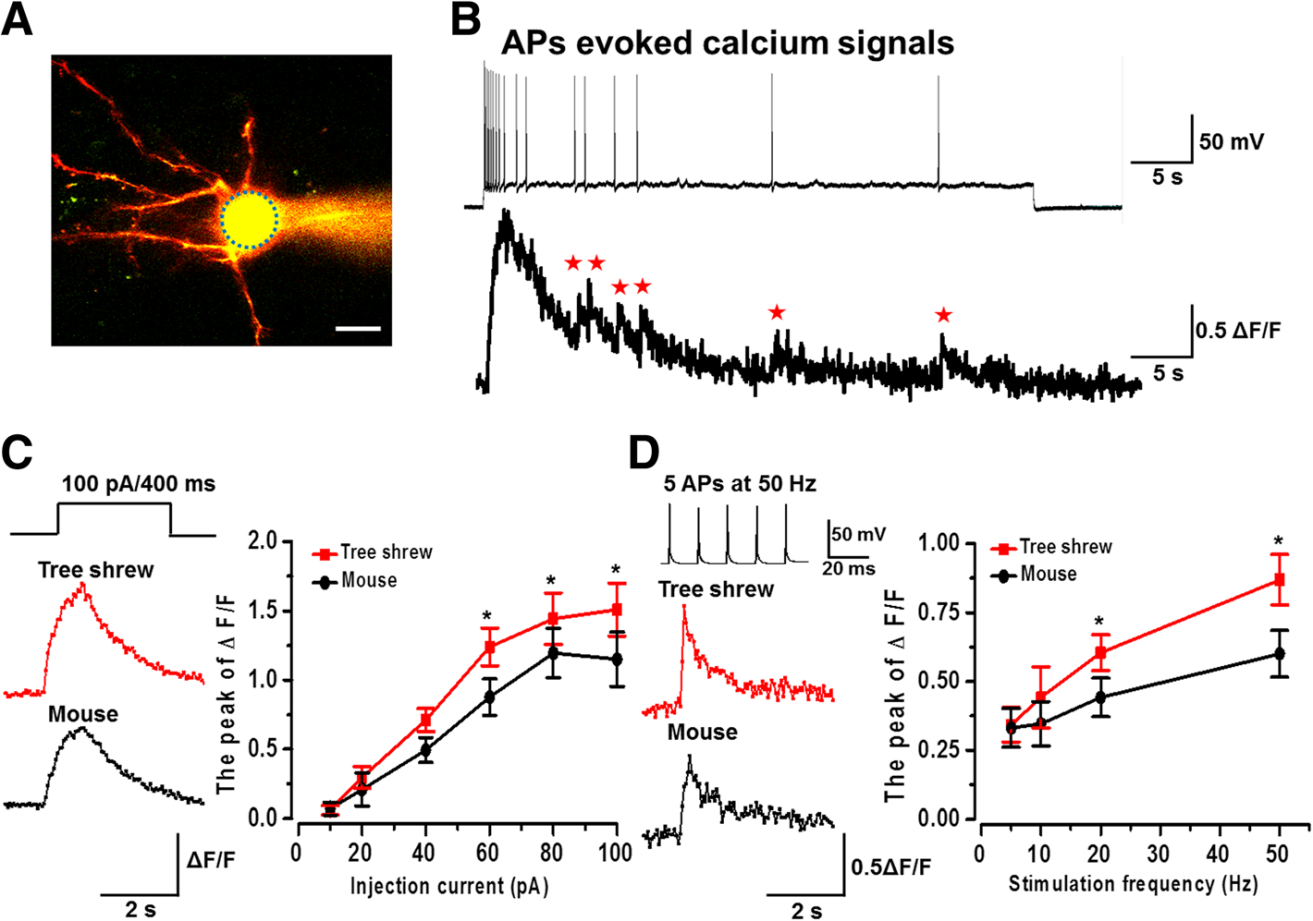
The global calcium signals in the ACC neurons of tree shrew. **a** Representative two-photon fluorescent photomicrograph of a patched pyramidal neuron loaded by Alexa 594 and Cal-520 in the ACC of tree shrew. Blue dashed circle indicated the scanned area on soma. **b** Injection of currents induced action potentials (APs) and related Ca^2+^ signals. Upper: representative traces of APs evoked by injection of 40 pA (50 s) current; Bottom: related waveforms of fluorescence changes (ΔF/F) of global calcium signals. **c** Calcium signals were increased in a stimulation ntensity dependent manner in tree shrew and mouse. Left: representative waveforms of fluorescence changes (ΔF/F) of Ca^2+^ signals evoked by injection of 100 pA (400 ms) current in tree shrew and mouse. Right: statistical data of stimulation intensity dependent Ca^2+^ signals in tree shrew (n = 6 neurons/3 tree shrews) and mouse (n = 7 neurons/3 mice). **d** Calcium signals were increased in a frequency dependent manner in tree shrew and mouse. Left: representative waveforms of fluorescence changes (ΔF/F) of Ca^2+^ signals evoked by five APs at 50 Hz in tree shrew and mouse. Right: statistical data of frequency dependent Ca^2+^ signals in tree shrew (*n* = 5 neurons/3 tree shrews) and mouse (n = 5 neurons/3 mice). Ca^2+^ signals (ΔF/F) were normalized to control values. *P < 0.05, error bars indicated SEM

Calcium signaling is critical for synaptic transmission and plasticity in the ACC [10, 26]. In the present study, by combining whole-cell patch recording and two-photon Ca^2+^ imaging observation, we recorded the global Ca^2+^ signals in the ACC pyramidal neurons of tree shrew. After 30 min diffusion of Alexa594 and Cal-520, the neuronal morphology was well labeled (Fig. 7a). Action potentials (APs) could be induced by injecting depolarizing currents into the soma of cells through the patch pipette. We found that global calcium transients were obviously observed when APs occurred (Fig. 7b). We then studied the Ca^2+^ signal responses for different stimulus intensities and frequencies in tree shrew neurons. The ΔF/F values of Ca^2+^ signals were both increased with intensities (10 to 100 pA) and frequencies (five APs at 5, 10, 20, and 50 Hz) dependent manners. Interesting, we found the Ca^2+^ signals were significantly larger in tree shrew than mouse (intensity: F_(1, 63)_ = 4.25, *P* < 0.05, *n* = 6 neurons/3 tree shrews and *n* = 7 neurons/3 mice; frequency: F_(1, 36)_ = 8.92, *P* < 0.01, *n* = 5 neurons/3 tree shrews and 5 neurons/3 mice; two-way ANOVA) (Fig. 7c and d).

### Intrinsic properties of the pyramidal neuron in the ACC of tree shrew

**Fig. 8:**
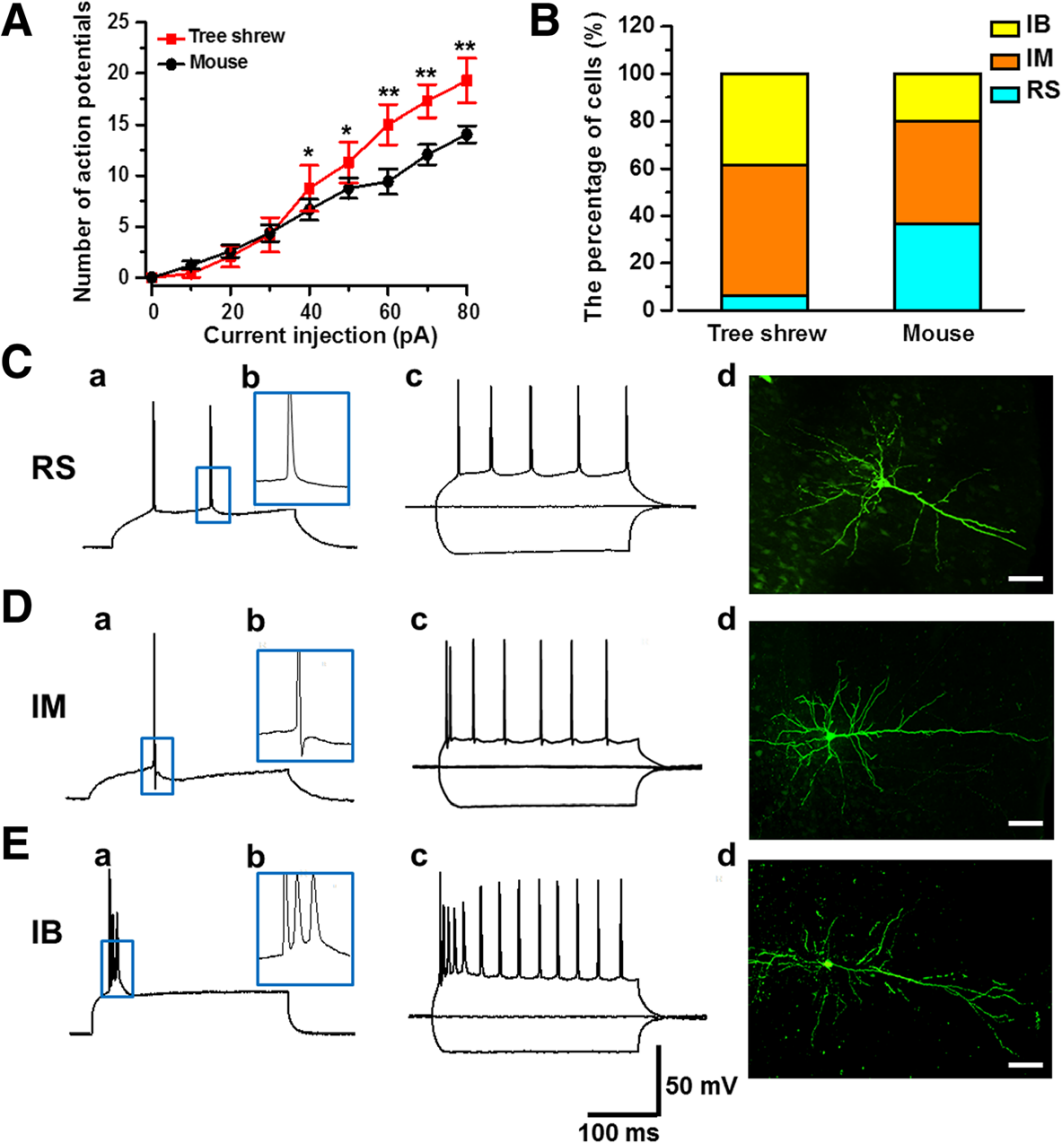
Morphological and intrinsic properties of pyramidal neurons in the ACC of tree shrew. **a** Averaged action potential numbers induced by step currents injection (400 ms, 10 pA per step) showed that the spike numbers of tree shrew neurons (*n* = 12 neurons/4 tree shrews) was larger than compared with mouse (*n* = 15 neurons/5 mice). **b** The percentage of three kinds of pyramidal neurons in tree shrew: regular spike (RS), intermediate (IM) and intrinsic bursting (IB) neurons (*n* = 65 neurons/22 tree shrews; *n* = 60 neurons/17 mice). **c-e** Electrophysiological and morphological properties of three kinds of pyramidal neurons in the ACC of tree shrew. A single current-clamp trace for the first spike induced by a series of intracellular current pulses (400 ms, 5 pA per step) (a). The blue frame in image (a) was enlarged in image (b). Superimposed current-clamp traces evoked by the current injections of −50, 0, +50 pA (c). Representative biocytin labeled profiles of recording pyramidal neurons as visualized with confocal laser scanning microscopy (d), scale bar: 50 μm

Our previous studies have shown that the intrinsic electrophysiological properties of the ACC pyramidal neurons in mice are important characteristics for neuronal excitability and can undergo dynamic changes according to sensory information inputs [37, 38]. We then examined the intrinsic properties of the ACC pyramidal neurons in tree shrews. As shown in Table 1, tree shrew neurons (*n* = 65 neurons/22 tree shrews) showed a larger membrane capacitance (Cm) (tree shrew: 131.48 ± 6.22 pF; mouse: 107.68 ± 4.90 pF; *P* < 0.05), smaller membrane resistance (Rm) (tree shrew: 263.56 ± 12.37 MΩ; mouse: 324.07 ± 31.52 MΩ; *P* < 0.05) and faster charge-discharge time (Tau) (tree shrew: 4.02 ± 0.14 ms; mouse: 4.92 ± 0.38 ms; *P* < 0.01), suggesting that the tree shrew pyramidal cells have a larger membrane surface and higher electrical responses capability. By analyzing single AP at the threshold, we found the half width (tree shrew: 1.25 ± 0.02 ms; mouse: 1.37 ± 0.04 ms; *P* < 0.01) and decay time (tree shrew: 1.09 ± 0.04 ms; mouse: 1.36 ± 0.08 ms; *P* < 0.01) were smaller in tree shrews than mice. The decay slope (tree shrew: −78.65 ± 2.72 mV/ms; mouse: −60.41 ± 4.69 mV/ms; *P* < 0.001) was larger in tree shrews as well. These results indicate that the spike of pyramidal cell is more narrow and sharp in tree shrews than mice. However, although the resting membrane potential (RMP) and the threshold membrane potential (*V*_threshold_) were not different, the rheobase (the minimum current required to evoke an AP) was higher in tree shrews compared to mice. Taken together, the present results suggest that, although a stronger current input is needed to initiate the spike (maybe due to the larger surface membrane and capacitance), pyramidal cells in tree shrews will spike more intensely than in mice. The hypothesis were further confirmed after injection of increased step current, in which the spike number of tree shrew neurons was not different with mouse neurons in face of weak inputs, but was significantly larger in face of stronger inputs (*n* = 12 neurons/4 tree shrews and *n* = 15 neurons/5 mice; F _(1, 217)_ = 38.94, P < 0.001, two-way ANOVA) (Fig. 8a).

**Table 1:** Summary of basal electrophysiological properties of pyramidal neurons in the ACC of tree shrew

	Tree shrew (n = 22)	Mouse (n = 17)	*t*-Test
Number of neurons	*n* = 65	*n* = 60	
Cm (pF)	131.48 ± 6.22	107.68 ± 4.90	*P* < 0.05
Rm (MΩ)	263.56 ± 12.37	324.07 ± 31.52	*P* < 0.05
Tau (ms)	4.02 ± 0.14	4.92 ± 0.38	*P* < 0.01
RMP (mV)	−71.46 ± 0.68	−70.36 ± 1.34	
*V*_threshold_ (mV)	−42.73 ± 0.87	−43.86 ± 0.85	
Rheobase (pA)	27.23 ± 1.91	17.60 ± 1.42	*P* < 0.01
Peak amplitude (mV)	98.47 ± 1.15	101.19 ± 1.51	
Time of peak (ms)	222.35 ± 8.51	233.60 ± 12.19	
Area (mV.ms)	124.30 ± 2.91	131.84 ± 4.39	
Half-width (ms)	1.25 ± 0.02	1.37 ± 0.04	*P* < 0.01
Rise time (ms)	0.63 ± 0.01	0.64 ± 0.02	
Rise slope (mV/ms)	130.85 ± 2.57	132.33 ± 3.39	
Decay time (ms)	1.09 ± 0.04	1.36 ± 0.08	*P* < 0.01
Decay slope (mV/ms)	−78.65 ± 2.72	−60.41 ± 4.69	*P* < 0.001
AHP peak (mV)	−9.67 ± 1.07	−7.75 ± 1.14	
ADP peak (mV)	9.05 ± 0.61	7.08 ± 1.36	

Values are means ± SEM

*RMP* Resting membrane potential, *AHP* Afterhyperpolarization, *ADP* Afterdepolarization

According to the action potential firing pattern, the pyramidal cells are classified into three groups: the regular spiking (RS) (AHP without ADP), intermediate (IM) (AHPs with ADP), and intrinsic bursting (IB) (the ADP will trigger bursting spikes) neurons. In our previous studies, IM and IB cells showed the higher membrane excitability than RS cell, and the population distribution of them were increased in neuropathic pain mice [38]. IB cells showed significantly greater firing frequencies than RS and IM cells after peripheral noxious pinch stimuli. In the present study, we found the ratio of IM and IB cells were higher, and RS cells were smaller in tree shrews than in mice (tree shrew: RS 6.1%, IM 55.4%, IB 38.5%, total *n* = 65 neurons/22 tree shrews; mouse: RS 36.7%, IM 43.3%, IB 20.0%, total *n* = 60 neurons/17 mice) (Fig. 8b and Table 1). For the morphological properties, we observed that all three kinds of neurons showed abundant basal dendrites and a prominent apical dendrite. Specifically, the apical dendrites of IB neuron sent forth mass branches which formed apical tufts. Taken together, these results further suggest that pyramidal cells in tree shrews are more active.

## Discussion

Cortical synaptic transmission and plasticity are critical for sensory and cognitive processes in mammals. However, there is limited information about cortical synaptic transmission and plasticity obtained from primate animal models. Recent cumulative evidence has shown that the tree shrew is a potentially useful primate-like animal model for human brain diseases [1–4]. In the present study, we investigated the excitatory synaptic transmission and intrinsic properties of pyramidal neurons in the ACC of adult tree shrews. We found that glutamate is the major excitatory transmitter for fast synaptic transmission. Both AMPA and KA receptors contribute to postsynaptic responses. As compared with excitatory responses recorded in mouse ACC, ACC in the tree shew show stronger excitatory transmission.

### Postsynaptic transmission in the tree shrew

Glutamatergic synaptic transmission plays important roles in both physiological and pathological conditions to play important roles in the ACC [9, 10]. In the current study, we found that AMPA receptor mediated responses were greater in tree shrews as compared with mice. However, there is no difference of NMDA receptor mediated EPSCs between tree shew and mouse ACC. This finding suggests that postsynaptic AMPA receptors are more effective in response to glutamate in tree shrew synapses. Future studies are clearly needed to explore a molecular basis for such difference.

Postsynaptic KA receptors contribute to fast synaptic responses in pain related cortical areas [17, 18, 39]. Here, we also detected a small fast excitatory synaptic response that is also mediated by KA receptors in the ACC of the tree shrew. Similar to AMPA receptor mediated responses, KA receptor mediated EPSCs are significantly greater than that in mouse ACC.

### Presynaptic transmitter release in the tree shrew

Both presynaptic and postsynaptic glutamate transmissions contribute to synaptic plasticity in the ACC [31, 35, 40]. In the present studies, we found that the frequencies of spontaneous/miniature EPSCs were smaller in tree shrews, suggesting that spontaneous release of glutamate in tree shrews is different from that of rodents. Furthermore, the ratio of PPF and the decay time for fast blockade of NMDA receptor mediated EPSCs are greater in tree shrews, which further indicate that presynaptic release of glutamate and plasticity may be different. It is interesting to note that enhanced postsynaptic responses and reduced spontaneous release of glutamate are features of tree shrew synapses in the ACC.

### Postsynaptic calcium signals and intrinsic properties of pyramidal neurons

Calcium signals are thought to be critical for synaptic transmission and plasticity in the ACC [9, 10, 13, 41]. By using two-photon Ca^2+^ imaging observation, our recent studies have characterized the properties of postsynaptic calcium signals in the pyramidal neurons of ACC in mice. We also reveal the dynamic change of Ca^2+^ ion in the induction phase of LTP in the ACC of mice [26]. In the current study, by using a similar method, we found that action potentials evoke significant Ca^2+^ signals in the ACC neurons of tree shrews and that the summation of calcium signals induced by repetitive stimulation is larger in tree shrew neurons as compared with mouse ACC.

We also identified three main types of pyramidal cells (RS, IM, and IB) in the ACC of adult tree shrews, which are similar with cell types reported in mouse ACC [37, 38]. We found that there are a higher proportion of IB and IM cells in tree shrews as compared with mice. This result indicates that neurons in the ACC are likely more excitable in tree shrews. We also found that tree shrew neurons showed higher initial firing frequency and neuronal excitability in the ACC. These results support the notion that the ACC of tree shrews are better developed on a functional level, which is similar with the suggestion about morphological properties of ACC neuron in tree shrew in our previous studies [5].

### Physiological and pathological implications

Animal models have been useful for the investigation of different physiological and pathological mechanisms of brain diseases. Cumulative studies have consistently indicated that ACC and related cortical areas play vital roles in many brain functions, including pain perception, fear memory, and anxiety [10, 19, 21]. Although human imaging studies provide strong evidence for ACC, the information on molecular and cellular mechanism in primate brain is generally lacking. The present study of tree shrew ACC provides a possible link between rodent ACCs and the human brain. We believe that the study of tree shrew brains, including the ACC area, will greatly improve our understanding of human brain mechanisms at molecular and synaptic levels, and help us to design better medicines and treatment for patients with different brain disorders in the future.

## Abbreviations

ACC: anterior cingulate cortex
ACs: adenylyl cyclases
ACSF: artificial cerebrospinal fluid
ADP: afterdepolarization
AHP: afterhyperpolarization;
AMPA: α-amino-3-hydroxy-5-methyl-4-isoxazole-propionic acid
AP5: D-2-amino-5-phosphonopentanoic acid
CNQX: 6-cyano-7-nitroquinoxaline-2, 3-dione
eEPSCs: evoked excitatory postsynaptic currents
KA: kainate
LTP: long-term potentiation
mEPSCs: miniature excitatory postsynaptic currents
NMDA: N-methyl-D-aspartic acid
PPF: paired-pulse facilitation
RMP: resting membrane potential
sEPSCs: spontaneous excitatory postsynaptic currents
